# Misuse, perceived risk, and safety issues of household insecticides: Qualitative findings from focus groups in Arequipa, Peru

**DOI:** 10.1371/journal.pntd.0009251

**Published:** 2021-05-06

**Authors:** Anika J. Larson, Valerie A. Paz-Soldán, Claudia Arevalo-Nieto, Joanna Brown, Carlos Condori-Pino, Michael Z. Levy, Ricardo Castillo-Neyra

**Affiliations:** 1 University of Washington, School of Medicine, Seattle, Washington, United States of America; 2 Department of Global Community Health and Behavioral Sciences, Tulane School of Public Health and Tropical Medicine, New Orleans, Louisiana, United States of America; 3 Zoonotic Disease Research Lab (LIEZ), One Health Unit, School of Public Health and Administration, Universidad Peruana Cayetano Heredia, Lima, Peru; 4 Department of Biostatistics, Epidemiology and Informatics, Perelman School of Medicine at University of Pennsylvania, Philadelphia, Pennsylvania, United States of America; National Center for Atmospheric Research, UNITED STATES

## Abstract

**Background:**

The current body of research on insecticide use in Peru deals primarily with application of insecticides offered through Ministry of Health-led campaigns against vector-borne disease. However, there is a gap in the literature regarding the individual use, choice and perceptions of insecticides which may influence uptake of public health-based vector control initiatives and contribute to the thousands of deaths annually from acute pesticide poisoning in Peru.

**Methods:**

Residents (n = 49) of the Alto Selva Alegre and CC districts of peri-urban Arequipa participated in seven focus group discussions (FGD). Using a FGD guide, two facilitators led the discussion and conducted a role-playing activity. this activity, participants insecticides (represented by printed photos of insecticides available locally) and pretended to “sell” the insecticides to other participants, including describing their qualities as though they were advertising the insecticide. The exercise was designed to elicit perceptions of currently available insecticides. The focus groups also included questions about participants’ preferences, use and experiences related to insecticides outside the context of this activity. Focus group content was transcribed, and qualitative data were analyzed with Atlas.ti and coded using an inductive process to generate major themes related to use and choice of insecticides, and perceived risks associated with insecticide use.

**Results:**

The perceived risks associated with insecticides included both short- and long-term health impacts, and safety for children emerged as a priority. However, in some cases insecticides were reportedly applied in high-risk ways including application of insecticides directly to children and bedding. Some participants attempted to reduce the risk of insecticide use with informal, potentially ineffective personal protective equipment and by timing application when household members were away. Valued insecticide characteristics, such as strength and effectiveness, were often associated with negative characteristics such as odor and health impacts. “Agropecuarios” (agricultural supply stores) were considered a trusted source of information about insecticides and their health risks.

**Conclusions:**

It is crucial to characterize misuse and perceptions of health impacts and risks of insecticides at the local level, as well as to find common themes and patterns across populations to inform national and regional programs to prevent acute insecticide poisoning and increase community participation in insecticide-based vector control campaigns. We detected risky practices and beliefs about personal protective equipment, risk indicators, and safety levels that could inform such preventive campaigns, as well as trusted information sources such as agricultural stores for partnerships in disseminating information.

## Author summary

In the region of Arequipa, Peru, there have been campaigns by the government to eliminate triatomine insects from local households, such as going door-to-door offering to spray homes with insecticides. This is because triatomine insects carry the life-threatening Chagas disease. However, many types of insects can cause problems in the home, and community members may be using other insecticide products in addition to the government spray campaigns. In this study, we asked household members in the Arequipa region what insecticide products they already used. We also did activities where study participants “advertised” insecticides to help figure out what was most important to community members about insecticides. We found that community members in Arequipa were using a wide variety of insecticides in the home in addition to the government spray campaigns. Many community members are concerned about the impact of insecticides on their health. We also learned that some community members were using insecticides in a way that could be dangerous–for example, using insecticides on children or in bedding, or not using proper safety equipment. We hope that future campaigns against triatomines include education, safety resources and working with households to make sure that the insecticides they use are safe and used correctly.

## Introduction

Household insecticide use and uptake of insecticide campaigns in Peru has been studied predominantly in response to serious health threats from vector-borne disease [1–6]. In Arequipa and surrounding areas where the current study was conducted, insecticide research efforts have focused particularly on addressing triatomine, or “kissing bug,” populations in response to Chagas disease [1,7–10]. One residential insecticide spray campaign in peri-urban Arequipa in 2012 reached only 66% of homes, and a study found that primary reported barriers included practical concerns such as spray times coinciding with work obligations and difficulties preparing the home, as well as concerns about the health impacts of insecticides and a perception that the campaign was not necessary [1]. Though unreported in that publication, some participants refused to have their houses sprayed giving the reason that they had already fumigated or used some form of vector control in their homes (i.e., the fumigation or vector control was either paid for to a private fumigation business or applied by a family member). This contributed to our concern about types and amounts of insecticide that might be in use in households in the region (and possibly used additionally to the indoor residual spraying (IRS) carried out by the vector control unit of the regional health office), leading to this study.

Studies of household insecticide use and community fumigation in Peru have historically focused on the chemical control of vector-borne disease targeting mosquitoes and triatomines [1–5,11,12]. However, previous studies in regions of Latin America threatened by vector-borne disease have identified high use of and expenditures on insecticide products such as aerosolized sprays in the home [13,14]. Household insecticide use may play an important role in the ecology of vector insects; a study of triatomine infestation in the Argentinian Chaco found that households with little to no domestic insecticide use were more likely to be infested with *Triatoma infestans* [15]. Studies worldwide have identified a wide variety of in-home strategies to combat insects including insecticide use, cleaning, and the use of physical barriers such as nets [16–18]. In Iquitos, Peru, in addition to participating in fumigation campaigns for mosquito control, many participants reported use of insecticide sprays and cleaning products indoors to combat mosquitoes as well as non-insecticide methods such as using petroleum or creoline to repel mosquitoes from their homes [16].

Household insecticides can present a serious health risk for household members, and children are at greater risk due to their developmental stage and behaviors [17–19]. Acute pesticide poisoning is a widespread and underreported problem in Latin America [17]. The Peruvian Ministry of Health documented a total of 2,489 deaths from acute pesticide poisoning in 2017 alone; 7.9% were classified as accidental and unrelated to occupational exposure to pesticides, and 8.5% occurred in homemakers and 3.2% infants [18]. Acute pesticide poisoning of children can be caused by household behaviors including storage of insecticides in unmarked containers and using insecticides directly on children [17].

Our study objective is to examine how household members in peri-urban Arequipa, Peru choose and use different insecticides in the home and explore what guides their decision-making process as they choose insecticides, using focus group discussions (FGDs). A greater understanding of perceptions and use of insecticides on the household level will help public health officials and policymakers to provide helpful and relevant tools and education to promote safe and effective insecticide use and combat vector-borne disease in vulnerable communities.

## Methods

### Ethics statement

Institutional Review Board approval was obtained from Universidad Peruana Cayetano Heredia (approval identification number: 65369), Tulane University (approval identification number: 14–606720), and University of Pennsylvania (approval identification number: 823736). Written consent to participate and to be audiotaped was obtained from all focus group participants.

### Study setting

**Fig 1:**
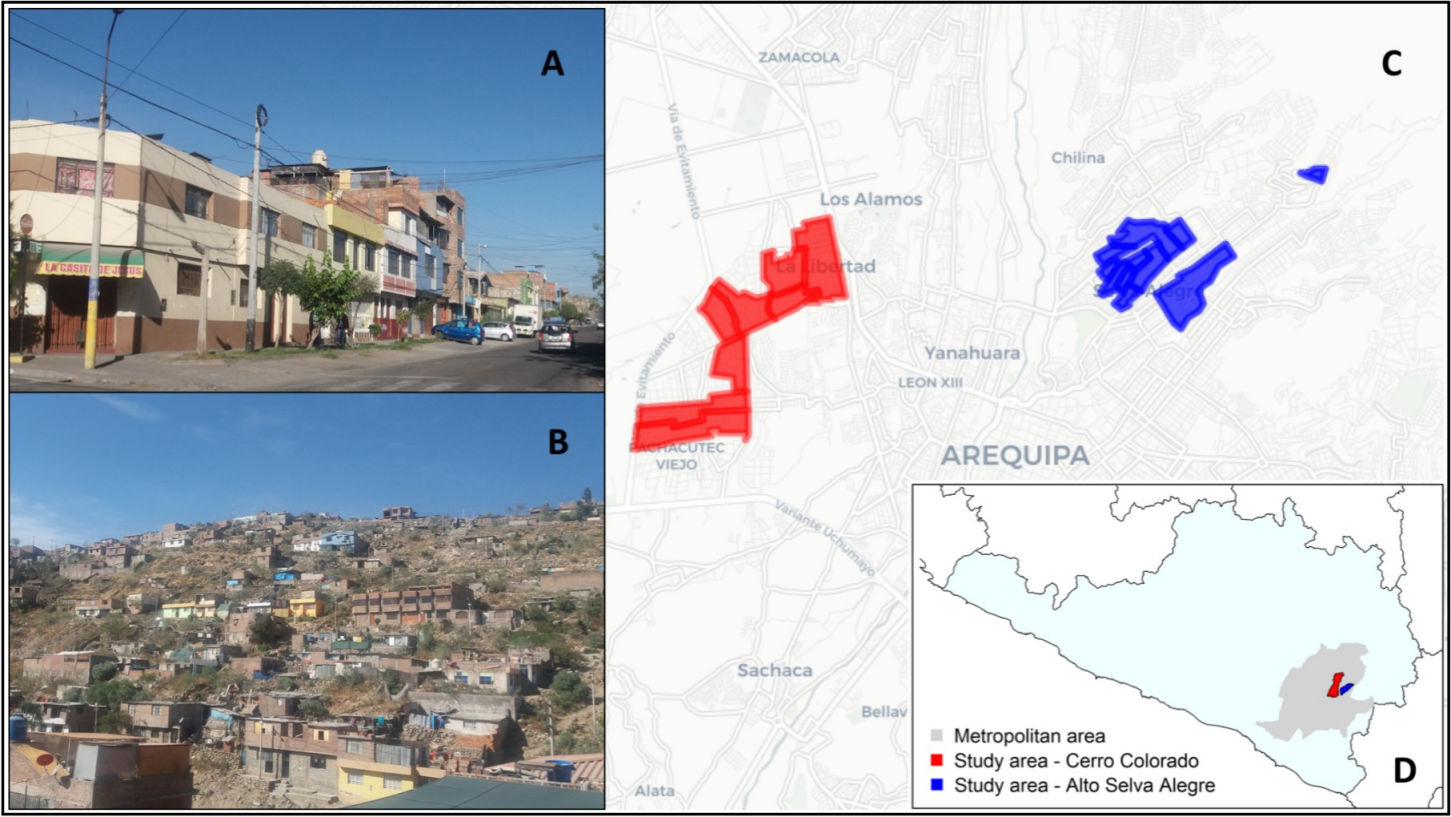
*Urban (A) and peri-urban communities (B) where FG were conducted*. *Distribution of localities where FG were conducted within districts Cerro Colorado and Alto Selva Alegre (C) and within the Arequipa Region (D)*. *Photographs for the attached figure taken by Laura Tamayo*, *and the base layer for the map is sourced with permission from the Database of Global Administrative Areas* [21].

The study was conducted in Alto Selva Alegre (ASA) (population for 2017: 85,870) and Cerro Colorado (CC) (population for 2017: 197,954), two districts located in the city and province of Arequipa. Arequipa Province is home to 1,080,635 people and Peru’s second largest city, Arequipa [20]. Our research team has worked in most districts of Arequipa, but due to recent government-led fumigation campaigns in ASA and CC for triatomines that can transmit *T*. *cruzi*, the parasite that causes Chagas disease, we selected these sites for the FGDs. The districts of Arequipa vary in human population size, house density, and socioeconomic status. They are formed by contiguous neighborhoods and this variation is usually associated with the level of urbanization of those neighborhoods, with lower levels or urbanization in peri-urban (peripheral) areas. ASA and Cerro Colorado span the gradient of urbanization. In our study, participants represented peri-urban residential areas of the city of Arequipa (Fig 1).

### Sampling strategy

Purposive sampling was used to select participants for seven focus group discussions (FGD). We invited all those who lived in ASA or CC and who had reported bed bug infestations in the past two years (approximately 15 individuals). We also recruited households with other types of insect infestations during the last 2 years (e.g. flies, cockroaches) by knocking on doors in houses 4 blocks around the bed bug infested house, but not within the same block. All participants were residents of the districts of ASA or CC. Recruitment criteria included having had a domestic insect infestation–whether bed bug or other—during the last two years, being over 18 years of age and willing to consent to the study.

### Recruitment approach

The recruitment strategies described in the previous section were applied until the quota (8 to 12 participants per focus group) was reached. In case people were unable to attend or chose not to participate in the focus groups, the team invited more than 12 individuals to ensure a minimum number of participants. An additional focus group was carried out due to low attendance in one group. Participants were explained the study goals by the recruitment team and who obtained written informed consent.

Participants were informed about the date and time of the FGD and confirmed their assistance. Arrangements were made to pick them up from their houses and take them to the FGD locations. Participants were given compensation for transportation back home.

### Study population

Forty-nine individuals, between 18 and 81 years of age, participated in seven FGDs (4 groups in ASA and 3 groups in CC) over four days. Seventy three percent of participants were female (n = 36). Most participants (all but 2 who did not respond or report a recent infestation) reported their most recent infestation in 2015 or 2016 with most recent infestations including spiders, flies, triatomines, ants, mosquitoes, cockroaches/beetles, moths, ticks, bedbugs, scorpions and livestock lice.

### Study design

A FGD guide was used to facilitate the data collection. The themes covered in the FGD guide included experiences with insect infestations in homes, information about use of insecticides and other pest control methods, decision-making and practices associated with the purchase and use of insecticide products, and risks perceptions about these products.

Prior to the FGD, the research team purchased different types of insecticides available in stores, in local markets, as well as sold by “agropecuarias” (small or medium size agricultural supply stores that sell manure, chemical fertilizer, seeds, as well as pesticides, and are usually located in the periphery of the city). During the FGD, participants were asked to break into groups of two, pick from the various insecticides set up on a table, and they were given 5–10 minutes to prepare a short marketing presentation about the product they chose, based on what they could read about on the product. The research team split up to be able to listen to the conversations of the various pairs. Then all FGD participants reconvened and they were asked to “sell” their product to the rest by making a short 1–2 minute presentation, while the research team noted what insecticide product elements were highlighted during these short presentations. This exercise enabled participants to share what characteristics they identified and valued in each product, as well as the meaning given to certain key words on packaging based on what words they chose to promote the product.

The FGDs were facilitated by a social scientist and a research assistant, with technical support of an infectious disease epidemiologist and a biologist with years of experience in insect vector control. There were two notetakers who are also part of the research team. All research team members involved in FG facilitation and support are Peruvian, and half of the team resides in Arequipa.

### Data management and analysis

With written informed consent, all FGD were digitally audio-recorded and transcribed; detailed notes were also taken throughout. An inductive coding process was used: we first grounded ourselves in the data to explore the topics that would emerge, not knowing what to expect [22]. Although the main themes being explored were established prior to the FGD, all subcodes were then developed based on the emerging themes. Focus group transcripts were imported into ATLAS.ti and coded into the four major themes: problems with insects in the home; choice of insecticides; use of insecticides; risks of the use of insecticides. As described, the subcodes were developed based on what emerged by theme. Main findings of interest were summarized using the coded material as well as the original transcripts. Relevant quotes are also presented by theme. Three of these themes are presented in the current manuscript; “Problems with Insects” is excluded and is published in a separate manuscript due to the volume of data.

## Results

Main themes that emerged in the focus group discussions are reported by sections. Key findings included safety/health, effectiveness, convenience and low odor as important factors in choosing insecticides, and “agropecuarios” (agricultural supply stores) as trusted sources of information (Table 1). There was widespread misuse of insecticides in the home, including informal fumigation, combining insecticides or mixing with other materials, direct use on children and animals, and inadequate use of safety equipment; participants reported symptoms consistent with acute pesticide toxicity when using insecticides as well as human and animal fatalities related to insecticide ingestion (Table 1). A table of themes and corresponding subcodes (Table 1) is included below.

**Table 1:** *Themes and Subcodes*.

Themes (Deductive)	Subcodes (Inductive)
**Section: Household Insecticide Use**	
**How and Where Insecticides are Used**	Home Use: Spray and powder insecticides are applied directly to home surfaces. Opinions were split whether they could be applied in the bed.
	Direct application: Insecticides as well as related products (flea shampoo) applied directly to children or pets.
	Preparation: Spray (convenient but strong) vs powder (easily accessible to children or pets).
	Preventive use: Insecticide can be applied preventively at intervals of weeks or months as informal fumigation.
	Government campaigns: Participants from both sites reported fumigation through formal government campaigns.
	Agricultural use: Participants used phosphine insecticide to protect agricultural products in stores, likely family businesses connected to the home.
**Frequency of Use**	Targeted insecticide use as needed: After first application, wait times ranged from half an hour to the following day.
	Routine insecticide spraying: Participants performed informal fumigation from every 2 weeks to every 2 months.
**Who Applies**	No clear distinction with the exception of adults rather than children.
**Applying Multiple Insecticides**	Combining insecticides: Done to combat infestations or address multiple insects at once.
	Risks: Combining insecticides was reported to increase health risks and risk of "resistance" to insecticides.
**Alternative Methods**	Cleaning and Hygiene: Maintaining a clean home and taking out trash were considered especially important for flies.
	Chemical methods: Alcohol was used for bedbugs, bleach for flies/ants, disinfectant, detergent or gasoline for ants, soap for plant insects, and mosquito repellant.
	Herbal/natural methods: Associated with older people, included eucalyptus, molle, muna, smoke (particularly of these herbs), ash, talc, hot water.
	Bed cleaning: For bedbugs, a multi-step process described multiple times including cleaning, sunning,washing, maybe insecticides/alcohol, sometimes burning and replacing bedding.
	Physical Methods: Fly screens, flyswatters, killing with brooms and shoes, fly tape, keeping home closed against mosquitoes.
**Section: PERCEIVED RISKS OF USE**	
**Health Risks of Insecticides**	Health impacts: Physical impacts of insecticides reported by participants included headaches, trouble breathing, hand pain, eyes burning, irritation, dizziness, vomiting, concern for "allergies" in children and death reported in an animal.
**Bad Experiences**	Accidental Ingestion: Accidental ingestion by people and animals was a concern and was reported in isolated cases, including a small child who drank bleach.
**Prevention of Insecticide Risks**	Timing: Applying when others (especially children) are out of the house; for spray insecticides, this includes ventilating on return.
	Storage: Keeping out of reach of children, such as storing in high places, child locks, etc.
	Proper use: Using as directed by package.
	Personal Protective Equipment (PPE): Participants described multiple types of often informal personal protective equipment, including gloves or bags on hands, masks or rags over the face, glasses, etc.
**Section: INSECTICIDE CHOICE**	
Criteria for Insecticide Selection *Also includes "Types of Insecticides—Contrasted"	Ease of use: Factors included whether it needed to be prepared, where spray was broadly considered more convenient and quick than the powdered insecticides.
	Safety: Factors affecting safety included whether it was easy to keep away from children and whether it lingered in the environment.
	Odor: Odor was discussed as a metric of strength or health impacts.
	Health: Participants advertised or described insecticides they personally used as "non-toxic".
	Strength/Effectiveness: Includes how well the product works, especially against certain insects like roaches, and how long a product lasts.
	Cost: Cost includes price, how long it lasts, cost-effectiveness. Cost was associated with quality and brand recognition.
	Familiarity: Participants used certain products out of habit or familiarity.
	Spray vs. Powder: In general sprays were faster, stronger, more expensive and toxic; powders were cheaper and risky for children (although the reverse was reported in one case).
Brands of Insecticides *Also includes "Types of Insecticides—Contrasted"	Strong and prestigious brands: Baygon, Raid and Sapolio
	"Non-toxic" and safer brands: Johnsons and Chica Verano were described either in advertisements or personal accounts as being less toxic.
**Relative Risks of Insecticides**	Determining risk: More potent, less familiar and cheaper types of insecticides are also considered to pose greater health risks.
	Packaging/form: Brightly packaged insecticides and those that can be grabbed by children (such as powder) were named as more risky
	Inherent risks: Participants also expressed that insecticides are inherently harmful.
**Specificity by Insect**	Insect specificity: It was important for some participants to match insecticide to insect and some requested it from the store for a specific insect, particularly for spiders, mosquitoes, flies and roaches.
	Broad-spectrum Insecticides: Certain well-known brands were considered potent, and insecticides that killed roaches were considered strong enough to kill other insects.
**Recommendations / Source of Information**	Reliable sources: "Agropecuarios" (feed stores), also called specialists, were the most trusted source.
	Veterinarians: Veterinarians were named as a source of advice as well as insecticides.
**Places to Purchase Insecticides**	"Agropecuarios" (feed stores): Most trusted source of insecticides.
	Markets or street vendors: Ambulatory and informal vendors were considered less reliable, since the product could be impure or expired.

### Proper and improper insecticide use

#### Where insecticides are used

Insecticides were used in numerous areas of the home including bedrooms, kitchens, bathrooms, and yards/patios. Powder insecticides, such as the popular “Chica Verano” (deltamethrin 5% soluble powder), were applied as indicated (dissolved in water) or pure as a powder (not the use indicated on the instructions) [23]. Spray insecticides were used as needed or for regular informal fumigation, ranging from every 2 weeks to every 2 months. A typical scenario was to spray within in the home, leave the homes closed for a period of time while children family members were away, and ventilate on return. Several participants also reported fumigation from the government spray campaigns against "chirimachas" (triatomines).

There was also a reports of insecticide spraying used directly on dining plates:

While working as a cook, we used it [insecticides] even on dishes… The lady told me “bring the Raid, quickly” and we didn’t even close the house. We sprayed it on the plates and then sat and talked… I asked her, “are we going to rinse the plates?” but she said that by the time we serve food it will have evaporated.”

-ASA

#### Frequency of insecticides use

Participants would wait from half an hour to the following day before repeating insecticide application for an ongoing infestation. Some participants reported that they would switch insecticides if the first did not work, combine the initial insecticide with another, use multiple targeted insecticides for multiple insect species, or mix insecticide with household cleaners. Others avoided combining insecticides due to concerns about health, decreased efficacy, or “resistance”.

*Facilitator: Have you ever used Chica Verano mixed with spray?*

Participant 1: Spray with Poett [brand of household cleaner].

*Facilitator: Spray with Poett. But have you ever used it, has anyone used insecticide combined with another?*

Participant 2: With "Folidol" [likely Methyl Parathion 20%].

-CC

#### How insecticides are used

Several cases of insecticide application directly to children and animals were reported. One participant used powdered deltamethrin 5% directly in her daughter’s hair against lice, tying a plastic bag over her daughter’s eyes as protection.

Participant: My little daughter [3 years old, but this was done when she was 2.5 years old approximately], with this [“Chica Verano” (deltamethrin 5%)] I have powdered her all, they have made me shampoo of this; ‘for the hair’ they said, ‘for fleas’, I put it on her … everything has disappeared, even the nits.

*Facilitator: Did you do it with dry powder or water?*

Participant: From here, I tied [her hair] like this and I put it as powder, if not it will get in her eyes and mouth.

-CC

*Facilitator: Has anyone else used anything else apart from Nopucid [lice shampoo]?*

Woman: My sister lives in Satipo [another city] and there are a lot [of lice] and I have seen her use an iron comb, I have also seen how she has reached despair and has put a powder for insects on the baby’s head.

-ASA

A few participants who sold or worked with bulk food products reported placing a vaporizing pill (a phosphine pellet or tablet designed to release insecticidal vapors for warehouse use) under products to keep insects away. For protection, participants who reported using phosphine used a piece of toilet paper to protect their skin and did not mention any gas monitoring. In one case, the participant (CC) reported using it in a warehouse overnight. Another participant (CC) left the vaporizing pill in a store during working hours; when asked if people in the same room with phosphine could be affected, he explained that you could not touch it but may have been unaware of the inhalation risk (or chose to ignore it).

“I wrap it in a piece of toilet paper and put it between the sacks; you put it under a few of them and the smell penetrates, everything goes away.”

-CC

Participants from both districts reported that they had or would use insecticides in beds, including children’s beds, but responses varied and participants from two focus groups agreed they would not immediately apply insecticide directly to a mattress, even if children were showing bug bites.

"I feel the fleas and run to buy and spray it. My children are sleeping and [I spray it] not too close to the face but near the feet…"

- ASA

Cleaning a bed for bedbugs was described in both regions and across multiple focus groups as a multi-step process that could involving taking the bedding and mattress out to the sun, washing bedding with bleach, using insecticide on the wooden bedframe, and spraying alcohol in the bed. As a last resort, participants also reported burning bedding and mattresses. One participant reported burning her own mattress in desperation to rid her bed of bedbugs after fumigating with “Baygon” every 2–3 days for three months.

Participant: The mattress, ah no, I burned the mattress.

*Facilitator: You have burned it?*

Participant: Yes, I have burned it, the bed, the mattress, everything because I was desperate. I burned everything.

-ASA

Other alternative methods of controlling insects including cleaning and disposing of trash, using non-insecticide chemicals such as alcohol, bleach, detergent, gasoline and soap, physical barriers or tools such as screens, flyswatters, water bags (placed on or hung over tables to “scare” flies away), and mosquito repellant. Herbal and natural methods of repelling insects included using herbs such as eucalyptus, mole, and muña (similar to mint); smoke, particularly of those herbs; ash, talc, and hot water.

“Well, in the house, since I’m an old lady, I burn eucalyptus.”

-CC

### Perceived risks of insecticide use

#### Health risks of insecticides

Participants reported that the short-term risks of insecticide exposure or ingestion included headaches, trouble breathing, pain in the hands, burning eyes, irritation, dizziness, vomiting, and even death of animals or people. Allergic reactions were a common concern, especially for children. Moreover, participants recognized a difference in individuals’ responses to insecticides–that the response to "Chica Verano" (deltamethrin 5%) might be fine for some, but that others might be sensitive to it.

Alarmingly, participants in both regions reported personal experiences with symptoms consistent with acute pesticide toxicity, including headache, vision changes and difficulty breathing.

Participant 1: Because if you stay a bit [in a room with insecticide], it harms you…

Participant 2: And your head hurts.

Participant 3: Your head hurts, and your vision [eyes] too.

-CC

Participant 1: When I use it, with that odor, my head hurts, it hurts my hand.

*Facilitator: It gives you a headache. Any worry about using the insecticide?*

Participant 2: Because of the vision.

*Facilitator: What happens with the vision?*

Participant 2: It burns.

-ASA

Several participants reported fatal incidents related to insecticide use. One participant described a friend who drank Malathion (organophosphate insecticide), thinking it was a medicine, and died. Another participant reported that after using a particular insecticide, her daughter became dizzy and her dog vomited; another reported that her puppy died from ingesting *“*Chica Verano*”*.

“I have had [bad experiences], with *Chica Verano*; trying to kill flies, I killed my puppy… And I took it to the veterinarian and even with a vet, I couldn’t save my puppy."

-CC

Children were considered inherently more vulnerable to the health risks of insecticides. Contributing factors included their behavior, such as picking up or swallowing insecticides, as well as the possibility of allergies and reactions to insecticides that they had never been exposed to before.

"I have had to fumigate in my work, but we were adults. But with children, we don’t know if they have allergies, because when they’re born you see the child but you don’t know what sickness they will have or what allergies they’re going to present, or what products will harm the child, so there’s no guarantee, you have to take them outside."

- ASA

One participant asked what toxicity meant and whether the odor and strength of an insecticide was a proxy of the toxicity. Several participants reported that all insecticides inherently presented a risk for health, because they were designed to be lethal to living things.

Participant: A question, for my knowledge, what is meant by toxic? The lady says it’s toxic, what should I understand?

*Facilitator: I don’t know, what do you understand it to mean?*

Participant: I don’t know if I have it right

*Facilitator: Yes, yes, go on*.

Participant: My dad used the baygon spray [insecticide spray made by “Baygon”], so he would protect himself, but it left a horrible odor, an odor that I couldn’t be around, so I couldn’t be there—it was a toxic atmosphere for me because it didn’t allow me to breathe for example… When I mix this product, it doesn’t have an odor, so from my point of view, is it less toxic or not toxic because I can be in the environment without using any type of protection?

*Facilitator: And how do you know you don’t need to use protection?*

Participant: Because it doesn’t say here either, “I let myself be guided by the smell,” by the strength.

-CC

#### Risk prevention during insecticide use

To reduce the risks associated with insecticides, some participants described using informal or homemade personal protective equipment; keeping insecticides stored in the home out of reach of children; timing their application of insecticides when family members were out of the house; and using insecticides as directed and seeking information on which insecticide to choose. However, risk prevention reporting was likely to be biased towards those that take precautions, and precautions described were frequently inadequate (e.g. the use of bags or handwashing for hand protection) and did not fully protect participants from acute exposure to insecticides.

Reported informal personal protective equipment included use of gloves or bags tied over one’s hands and glasses, and masks or rags over the face. Another participant explained that he did not use gloves for one insecticide because it was a powder, and his decision regarding need for insecticide protection was determined by the strength of the odor: if he could stand to be in the environment with the insecticide he did not need protection. One reported that she thought the majority of people did not use these precautions.

Participant: I put bags [on my hands]. If I don’t have any, I just wash my hands—water with soap–and I put on my glasses.

*Facilitator: Glasses?*

Participant: Because I have bad eyesight, a mask.

*Facilitator: You use a mask?*

Participant: When you don’t have money, what are you going to do?

-CC

To prevent harm to children, some participants described keeping insecticides in high places or in hidden areas of the house, as well as spraying when children were out of the house. However, one participant said that her daughter would touch the insecticides regardless of precautions. Another woman described her daughter touching walls wet with insecticides after a government-led fumigation campaign.

"You have to prepare chica verano, it takes a little more time, of course it’s good too! Because if there are small children, we are freed from them being intoxicated… I have a little girl that is very naughty. In order to sprinkle this, I have her by my side and I go with her. But all the same she touches because she is very curious. Children don’t warn you the moment they’re going to do something, in one second, boom [they are up to something]."

- ASA

Some participants reported knowing how to use insecticides safely by following the directions on an insecticide and reading warning labels. Others said that before using a product, they would seek out information, usually from trusted sources such as agricultural stores. However, during the focus groups facilitators noted that few participants could find the warning instructions in the actual bottles, cans and bags of commercial insecticide provided.

### Choosing insecticides

#### Ease of use

Spray and powder insecticides were the two major forms of insecticides discussed. Spray insecticides were considered quicker and more convenient, since they did not require preparation and could be used immediately on seeing an insect, while powder insecticides had to be mixed and prepared in order to convert them into a spray.

*Facilitator: But do you use the one that is a spray or sometimes you use another one*.

Participant: No! Just the spray.

*Facilitator: And why do you always use the spray?*

Participant: For me it is more comfortable and more practical.

-CC

#### Safety

Three themes emerged associated to insecticide safety: stronger insecticides were considered more harmful to health; the ease of keeping the insecticide away from children (including a safety lock and whether the packaging was colorful and enticing); and permanence in the environment after use. Powdered insecticides were referred to as a safety risk because they remain in the home and can be found by children in contrast to spray that evaporates. However, *“*Chica Verano,*”* a powder insecticide used by participants in both regions, was referred to as non-toxic by multiple participants during the advertising group activity and one participant used it directly on her daughter as discussed in section 3.1.3.

*Facilitator: [In the activity] we saw that many people preferred the sprays over the bags of powder. Why would they choose the sprays more than the powders?*

Participant 1: For the practicality of the sale.

Participant 2: Practical. When we have children, we don’t use the powder, they can grab it. In contrast, with the spray they go to school and we take advantage and spray it.

-CC

#### Strength

The strength or effectiveness of an insecticide was an important factor and was associated by many respondents to the odor: the stronger the odor, the stronger the insecticide and the more harmful to people and long-lasting it would be. In general, “Baygon”, “Raid”, and "Sapolio" were perceived as the most potent and strongest brands. Some participants purchased insecticides to the insects they were designed to kill, especially for spiders, mosquitoes, flies, and roaches, but certain insecticides were considered “broad-spectrum” and it was agreed that if an insecticide could kill cockroaches, it could kill any bug.

#### Cost

Lower priced insecticides were generally associated with lower quality, including the possibility that insecticides were expired or not genuine products or were more harmful. One participant noted both that all cheap products were harmful, but also that high prices or brand names were not necessarily an indication that a product worked.

"Maybe because it’s more economical, so of course I say ‘No!’ If it’s cheap, it is not so effective, I save a little more and I buy a better one."

-CC

#### Sources of insecticides and information

The most trusted sources of information and insecticides were “agropecuarias,” or agricultural feed stores. Agropecuarias gave advice and recommendations regarding the most appropriate insecticide, as well as advising on possible health impacts. Street vendors were considered cheap sources of insecticides but possibly unreliable; participants reported that the insecticides could be “bamba” (knockoffs) or expired.

*Facilitator: Have you gone to the street sellers or agriculture stores? Have you asked: what do you recommend to me, what can I use, what is best, what is most dangerous?*

Participant 1: We guide ourselves by the labels that say ‘for flies, cockroaches’. If we want for rats, we go and look, but like they say, it is better to go to an agriculturist and they will give us better information.

Participant 2: I once went and they asked me if anyone had allergies in the house. Do you have young children they asked? Because they say you can’t use it if anyone in your family gets welts. I told them no.

-ASA

“We go to the market, we see that there are a ton of insecticide vendors. Those little packets, sometimes there are knockoffs, they cost less. It costs S/.1.00, S/.1.50 [US$0.30–0.50]. In order to try it out, I buy it, but they are not effective."

-ASA

## Discussion

The findings of this study raise significant concerns for insecticide safety in peri-urban Arequipa, including the availability of off-label pesticides and misuse within homes and workplaces. Home storage of pesticides, exposure to indoor pesticides, and direct contact with pesticides (including direct use of deltamethrin 5% on a child) were all identified in this study (Table 1). These are known risk factors for various health impacts among children including acute pesticide poisoning, childhood cancers and developmental toxicity [17,24,25]; a 5-year-old in Peru died in 2010 after drinking an unmarked bottle of "Chica Verano" (deltamethrin 5%) [26]. Participants reported symptoms consistent with acute pesticide poisoning (headache, vision changes, and difficulty breathing) [27] when using insecticides as well as human and animal fatalities related to insecticide ingestion.

Participants’ accounts of the availability and incorrect use of pesticides for use in households and small businesses is concerning, especially in the context of family-owned businesses where children may be exposed to pesticides used to protect agricultural products and in conjunction with high annual incidence of pesticide poisoning in Peru and Latin America as a whole [17,18]. Safety measures were generally informal and included makeshift hand and face coverings in place of personal protective equipment. A pellet form of phosphine insecticide was used by participants with only paper as protective equipment to fumigate stored food products, with a participant commenting incorrectly that you could be in the same room with it as long as you did not touch it.

The ultimate goal for reduction of pesticide poisoning from a global health perspective should be the phasing out of the WHO Class I and II pesticides (deltamethrin, for example) supported by the introduction of safe alternatives [28,29]. The health benefits of insecticide use against disease vectors such as mosquitoes and triatomines should be weighed relative to the possibility of misuse and resistance [30,31]. Research in the Argentine Chaco indicated that households with domestic insecticide use were less likely to be infested with triatomines prior to the initiation of a formal spray campaign, indicating that household insecticide use plays an important role in vector control [15]. However, there is also evidence that the use of household insecticides contributes to health impacts for family members and insecticide resistance [17,32,33]. Of the major household brands identified in the current study many include pyrethroid insecticides, a class that is widely used in the Americas for control of Chagas disease [31] and presents a risk for development of resistance as well as acute health impacts [31,34,35].

These risk-benefit analyses are further complicated since both insects and insecticides contribute to health risks. For example, one impact of insecticides on children reported by participants was “allergy”; a study in northern Mexico found that there was an association between insecticide use in the home and allergies in children, but noted that this association was likely due to the presence of insects which contributed to allergies in children and were independently associated with higher insecticide use [36]. In addition, insects such as bed bugs or cockroaches are difficult to quantify in terms of health risks but represent practical and psychological burdens to community members in our study [37].

Peruvian governmental organizations including SENASA (El Servicio Nacional de Sanidad Agraria) regulate and monitor the sale and use of pesticides and continue to refine the types of pesticides permitted for use; however, there are ongoing challenges with expired, contraband, and adulterated pesticides as well as unsafe use [38–43]. This is consistent with the practices reported in our study of purchasing insecticides from “ambulatory” vendors which may expose household members to mislabeled insecticides as well as bypassing product regulations, as well as use of insecticides with minimal or informal safety equipment. Future campaigns should build awareness of safe use of household insecticides and prioritize community values such as safety, convenience, health and low odor of insecticides for household members to develop campaigns that can be safely incorporated into current household practices. Strategies such as integrated vector control can be used to maximize the long-term benefits of insecticides against vector-borne disease while minimizing risk [44,45].

Previous campaigns to promote safe pesticide use in Latin America have demonstrated the feasibility of partnering with local governmental and private organizations to promote safe use of insecticide as well as alternatives; the *EcoSalud II* program in Ecuador partnered with local governments to sponsor a local store to offer alternatives to riskier pesticides, such as insect traps [46]. Participants in our study reported that "agropecuarias" or agricultural supply stores are a reliable source of information about health and use of insecticides. Possible next steps for safe vector control campaigns in the Arequipa region might include integrating a partnership with local “agropecuario” stores alongside governmental spray campaigns to offer the safest known options for insect management along with educational materials and safety equipment. High-quality Spanish language educational materials regarding safe insecticide use in the home [47,48] as well as Peruvian educational materials on safe use of pesticides [49,50] are available on-line and could be adapted for households in the Arequipa region. We support and encourage future research in vector control campaigns incorporating education and support for local households in safe use of household insecticides. While income was not directly explored in this study, it is likely that resource limitations contributed to use of off-label insecticides as well as use of minimal or informal personal protective equipment and should be addressed in future research.

Significant limitations to this study include the limited number of participants, which restrict the generalizability of these findings to broader populations or a more in-depth or stratified examination of themes. However, the aim of this qualitative study was to explore issues associated with insecticide use, from improper use to overuse, and the study was not meant to be representative nor generalizable. While we found concerning reports of insecticide misuse among the participants in our study, and while it is not clear how widespread such practices are in the community, the fact that this issue emerged across FGDs reveals the relevance of this issue to many and the importance of further research and community education on this topic. The use of guiding themes and prompts in the focus groups enabled us to hold focused discussions regarding use and safety of insecticides within a timeframe of 1.5–2 hours, but introduces the potential for bias by facilitator guidance; we minimized this by relying on experienced facilitators and a previously determined set of open-ended topics. In addition, the use of our novel insecticide “advertisement” activity may have prompted participants to exaggeratereport positive aspects of insecticides that did not correspond to actual beliefs; whenever possible, we relied on accounts of how insecticides were used by participants to characterize perceptions of the actual benefits and health impacts of insecticides.

## Conclusions

Misuse of insecticides is common in households and small businesses in Arequipa, Peru, subjecting those exposed to toxic levels of these products based on their descriptions of experiences when using these. This is consistent with previous literature in Peru and worldwide demonstrating a significant health and resistance burden of insecticide misuse despite government regulation and monitoring. Our study provides more context into the motivations and methods of insecticide use on the household level, including the need to address a wide variety of vector insects, as well as nuisance insects, and the availability of cheap, off-label insecticides. This can be addressed in the context of vector control through integrated vector control campaigns and community partnerships with government, businesses, and community members. Vector control campaigns utilizing insecticides in this region should recognize that household insecticide use may already include informal fumigation and, in some cases, direct application to animals and children without adequate protection. Campaigns should also be cognizant of concerns about both immediate safety and health impacts of insecticide use and a promising direction for future research and campaign development may be partnerships with trusted sources, such as "agropecuarias," to disseminate information on insecticides and their safe and correct use.
